# Phase- and season-dependent changes in social behaviour in cyclic vole populations

**DOI:** 10.1186/s12898-019-0222-3

**Published:** 2019-01-25

**Authors:** Kaja Johnsen, Olivier Devineau, Harry P. Andreassen

**Affiliations:** Faculty of Applied Ecology, Agricultural Science and Biotechnology, Inland Norway University of Applied Sciences, 2480 Koppang, Norway

**Keywords:** Extrinsic, Intrinsic, Myodes, Population cycles, Territoriality

## Abstract

**Background:**

Social behaviour has been linked to hypotheses explaining multiannual population cycles of small rodents. In this paper we aimed to test empirically that the degree of space sharing among adult breeding female voles is higher during the increase phase than in the crash phase, and that the degree of sociality is positively related to population growth rate as suggested by Lambin and Krebs (Oikos 61:126–132, 1991) and Andreassen et al. (Oikos 122:507–515, 2013). We followed 24 natural bank vole *Myodes glareolus* populations over an area of 113 km^2^ by monthly live trapping throughout a complete population cycle of three summers and two winters.

**Results:**

Using spatially explicit capture-recapture models, we modelled the overlap in adult female home ranges and total population growth rate per season. We identified an increase phase before and during the peak density observation and a crash phase following the peak. Female home range overlap were seasonal- and phase-dependent, while population growth rate was associated with season and female home range overlap. High female home range overlap in the increase phase corresponded to a high population growth rate.

**Conclusions:**

We suggest that intrinsic social behaviour plays a key role in the increase phase of vole population cycles, as social behaviour leads to an increased growth rate, whereas extrinsic factors (predation and/or food) initiate the crash phase. Our results are consistent with those of other studies in a variety of small rodent species.

## Background

Social interactions linked to territoriality are among many factors contributing to the shaping of mammalian population dynamics [1–8]. There are several definitions of territoriality [9], but it is most commonly defined as dominance through social interactions over a specific geographic area [10]. For small secretive species in which it is hard to determine whether a territory is actively defended or not, territoriality is generally defined as an exclusive use of space [11]. Regardless of whether active defence occurs or not, the result of territorial behaviour is the exclusive use of space, and hence territoriality is a factor which can limit population size.

Mammals show great flexibility in territorial behaviour. Within the same species there may be both territorial and non-territorial individuals, and the same individuals may even change between territorial and non-territorial behaviour [12–14]. This flexibility may be an adaptation to variations in the spatiotemporal environment in which the individuals live [15, 16]. For instance, social behaviour may provide benefits by increasing protection against predators, creating mating opportunities, reducing the risk of infanticide or by increasing success in finding and maintaining access to resources [2, 17]. At the same time, it can be costly due to increased competition for resources and mating opportunities, or increased risks of disease transmission or conspicuousness to predators [2, 18–20].

Social behaviour has been linked to many hypotheses, e.g. kin selection, explaining multiannual population cycles in the northern hemisphere [6, 7, 21–25]. Lambin and Krebs [7] described how changes in relatedness among female vole and lemming populations affected population growth rate; high degree of relatedness caused more overlap between female home ranges, higher reproduction and population peaks, while low relatedness lead to competition for territories and population declines. Andreassen et al. [5] provided a multifactorial model describing how the interaction between social behaviour (an intrinsic factor) and extrinsic factors (e.g. predation) could contribute to shaping the population dynamics of small rodents. In an individual based modelling exercise, it was later shown that this model could lead to population cycles with similar attributes (e.g. periodicity and amplitude) to those found in natural vole populations in Fennoscandia [26]. The model described phase-specific demographic responses associated with changes in sociality and extrinsic factors. The increase phase (1) consisted of highly stable social groups (possibly matriarchies as described by Lambin and Krebs [7]) with high survival and high reproductive rates. Amicable behaviour among normally territorial individuals was presumed to result from abundant, possibly patchily distributed, resources; the crash phase [2] was characterized by both a disruption of the social system and low recruitment and low survival. This disruption of the social system was expected to be mediated by increased predation rates, specifically of dominant individuals, followed by infanticide and increased movements of other individuals [5]. In particular it has been shown that social females have a higher mortality rate if they are exposed to the infanticide of pups [27]. Hence, the model suggests that the increase phase should be characterized by less territorial behaviour than the crash phase.

In the bank vole, *Myodes glareolus*, adult females have mutually exclusive home ranges [28], whereas adult males have large home ranges that overlap extensively [29]. It has been hypothesized that food availability is one of the main causes of territoriality in female small rodents [13, 30, 31]. Females relying on sparse and slowly renewed food resources are expected to defend territories covering important food patches [11]. Alternatively, it has been suggested that females defend territories to prevent infanticide [12, 32–34]. However, territoriality of female bank voles is quite flexible. Rémy et al. [35] found that a clumped and highly predictable food source increased sociality, which led to a positive effect on the population growth rate through higher success in producing weaned offspring. Other studies have shown that bank voles are less territorial during winter [36], when communal living is expected to be beneficial due to enhanced thermoregulation [37]. In this study, we monitored up to 24 natural bank vole populations monthly for over 2 years, through the increase and crash phase of a population cycle, to empirically test the assumed phase dependent changes in sociality of the multifactorial model of Andreassen et al. [5] and the kin selection model by Lambin and Krebs [7]. We used female home range overlap as a proxy for sociality, where a higher degree of home range overlap among females indicates a higher degree of social tolerance. We predicted an association between social behaviour and the phase of the population cycle, with greater sociality, i.e. higher home range overlap, among female bank voles during the increase phase than during the crash phase.

## Materials and methods

### Study area

The study took place from June 2013 to August 2015 in Stor-Elvdal municipality, Southeast Norway (61°N, 11°E). The study area was a typical boreal forest dominated by Norway spruce (*Picea abies*) and Scotch pine (*Pinus sylvestris*), with bilberry (*Vaccinium myrtillus*) in the field layer and mosses (*e.g. Pleurozium schreberi*) in the ground layer. The climate was continental with relatively dry weather, large diurnal variation in temperature, warm summers and cold winters [38]. Snow cover normally lasts from December to April. The region experienced peaks in vole population density during the breeding seasons of the years 2007, 2011, 2014 and 2017 (Current study, and unpublished data from Inland Norway University of Applied Sciences).

### Trapping procedure

**Fig. 1:**
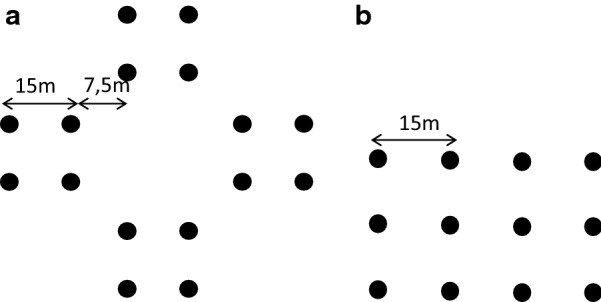
Trapping grid design. **a** Shows the main, cross-shaped design with 16 traps, and **b** the alternative design used when the main design did not encompass any suitable vole habitat, with 12 traps

Voles were caught on 60 m × 60 m grids consisting of 16 Ugglan multiple capture live traps (Granab, Sweden) arranged in a cross pattern, with 15 m spacing (Fig. 1a), along three elevation transects (Mykleby, Gåla and Evenstad). The transects ranged from 267 to 801 meters above sea level (mean altitude Mykleby: 537 masl., Gåla: 662 masl., Evenstad: 429 masl.). Each transect consisted of 8 trapping grids (n = 24). Grids were separated by at least 500 m. They were located in typical bank vole habitat, in mature forest areas with a bilberry-dominated field layer [39, 40]. Where cross-shaped grids did not encompass suitable habitat (n = 5), grids were arranged in a linear shape with 9 to 12 traps (Fig. 1b). Traps were placed in runways or close to holes with potential vole activity. All traps were marked with a stick and a ribbon in the closest tree and remained in the same place throughout the study. Between trapping sessions, voles could use the traps as part of their runway system. During winter, each trap was placed inside a plywood-box (30 cm × 30 cm × 40 cm) with no floor, making it possible for voles to move freely in and out of the box, and with a lid to prevent the traps from being covered with snow. The boxes were removed in spring when the snow melted around them. During each monthly trapping session capture-recapture trapping was carried out for three nights, i.e. traps were checked every morning and evening yielding a total of 6 secondary trapping sessions. We trapped from June 2013 to August 2015, with the exception of September 2014. During winter we reduced the number of trapping grids to 14 due to more time consuming trapping in winter. We also lost some trap nights due to extreme cold (− 20 °C), and some trapping grids due to heavy snow concealing the traps (Table 1).

**Table 1:** Trapping history

	Transects	Number of trapping grids	Number of secondary occasions	Tot number of captures	Tot number of adult female captures
June 13	3	24	5	72	39
July 13	3	24	5	328	115
August 13	3	24	6	707	254
September 13	3	24	6	769	285
October 13	2	16	6	1014	300
November 13	1	7	6	301	51
December 13	2	14	6	484	68
January 14	2	6	3	39	1
February 14	2	13	6	178	14
March 14	2	14	6	126	18
April 14	2	14	6	211	79
May 14	2	14	6	318	118
June 14	3	24	6	497	218
July 14	3	24	6	713	214
August 14	3	24	6	961	203
September 14	0	0	0	0	0
October 14	3	24	6	818	109
November 14	3	24	6	472	44
December 14	2	14	6	197	6
January 15	2	14	6	142	0
February 15	2	14	6	114	0
March 15	2	14	6	54	0
April 15	2	14	6	46	0
May 15	2	14	6	1	1
June 15	3	24	6	11	4
July 15	3	24	6	13	1
August 15	3	24	6	11	0

The number of secondary occasions express the number of times traps were checked during a trapping session (per month)

We baited the traps with oats and carrots, and we added sawdust in the cold period to absorb urine, preventing it from freezing onto the voles, and to help keep them warm. All voles were individually marked with a pit-tag (1.25 × 7 mm ID-100VB Nano Transponder) and sexed, weighed to the nearest gram and checked for reproductive status (mature if open vagina in females and scrotal testicles in males). We used a basic LID-560 Pocket Reader to read previous tags.

### Data analyses

#### Season and population phases

**Fig. 2:**
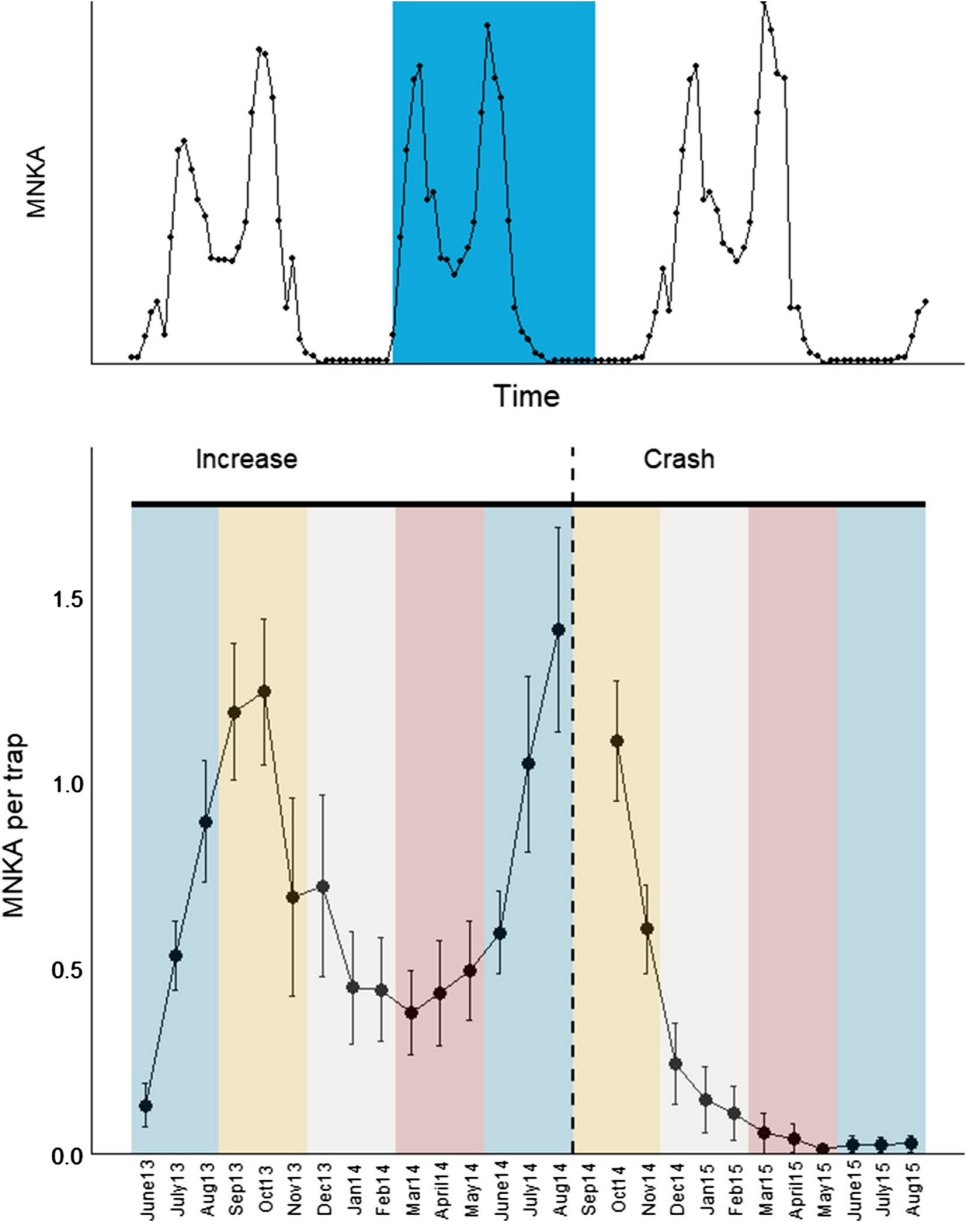
Top: a simulation with three peak years illustrating the cycle phase in the present paper in blue. Bottom: minimum number known to be alive (MNKA) per trap per month from June 2013 to August 2015 (missing trapping in September 2014 denoted with NA). We used these estimates to define the increase and decrease phases of the cycle for further analyses. Seasons are shown with colours: blue = summer, orange = fall, white = winter, red = spring

Our study consisted of trapping data from a complete population cycle, including an increase, peak, crash and low phase. We used the minimum number of bank voles known to be alive (MNKA) to give a preliminary monthly description of the population trajectory (Fig. 2) which we then used to identify the cyclic phases. MNKA gives us a more detailed (monthly) population trajectory than the density estimates (described below) gives us. Both the summers of 2013 and 2014 had high population densities with a winter decrease in between. Hence, we chose to consider the whole June 2013–August 2014 period as the *increase* phase, with a peak in August 2014. We considered the period starting in September 2014 as the *crash* phase of the cycle. The population crashed and reached the low phase in late winter 2014/2015.

We defined the seasons as summer: June–August; fall: September–November; winter: December–February; and spring: March–May (Fig. 2).

#### Spatially explicit capture-recapture models

Spatially explicit capture-recapture (SECR) models assume that each individual has an activity centre, and that the capture probability is a function of the distance between the trap and the individual’s activity centre. We estimated the density (D) and the spatial scale of detection sigma (σ) for each trapping grid and season using SECR models [41, 42]. We fitted the models using the secr package [43] in the statistical software R [44]. The parameter σ describes the relationship between the detection probability (g0) and the distance between a trap and an animal activity centre; it can be understood as a metric of home range size, where the spatial scale of detection (σ) increases with the home range size [8, 41]. To estimate the detection probability we used a half-normal detection function and a spatial buffer of 50 m. The model parameters (D, g0, σ) were set to be dependent on season rather than month to obtain long enough trapping histories for the SECR-modelling. We had too few recaptures to include any covariates in the models, and consequently there were no need for model selection.

Efford et al. [41] suggested re-parameterizing the models using k = σ √D, which is an expression of home range overlap between individuals. Consequently, if we assume σ to be a circular bivariate normal home range, it is possible to estimate the number of individuals within a certain percentage isopleth of the home range. The relationship between home range size and density can then be expressed as σ = k/√D or as a linear regression equation: log(σ) = log(k) + β log(√D). If overlap is constant among populations [i.e. the intercept log(k)], the slope β is assumed to be − 1/2 as home range size is halved when density is doubled [41].

We were not able to estimate k for adult females at the grid level, because the female population size was too low. We therefore ran the linear regression described above between log(σ) and density, where we weighted sigma by 1/SE^2^ [45], and obtained the residuals. We used these residuals as an index of home range overlap, with negative (or positive) values indicating less (or more) overlap than expected from the population density. After November 2014 there were too few captures to estimate any parameter of the population.

We estimated home range overlap among adult females only as we were interested in the changes in sociality in the territorial part of the population, while we estimated population density for the whole trappable part of the population.

#### Linear mixed models

We analysed the factors influencing home range overlap with linear mixed models using phase and season as fixed effects and trapping grid as a random effect. We calculated the seasonal population growth rate (D_t_/D_t−1_) where *t* was the 3-month seasonal period, and analysed it using linear mixed models with phase, season and female home range overlap as fixed effects and trapping grid as the random effect.

We performed model selection based on Akaike’s information criterion corrected for small sample sizes (AICc). All analyses were done using R [44], with the package lme4 [46].

## Results

**Fig. 3:**
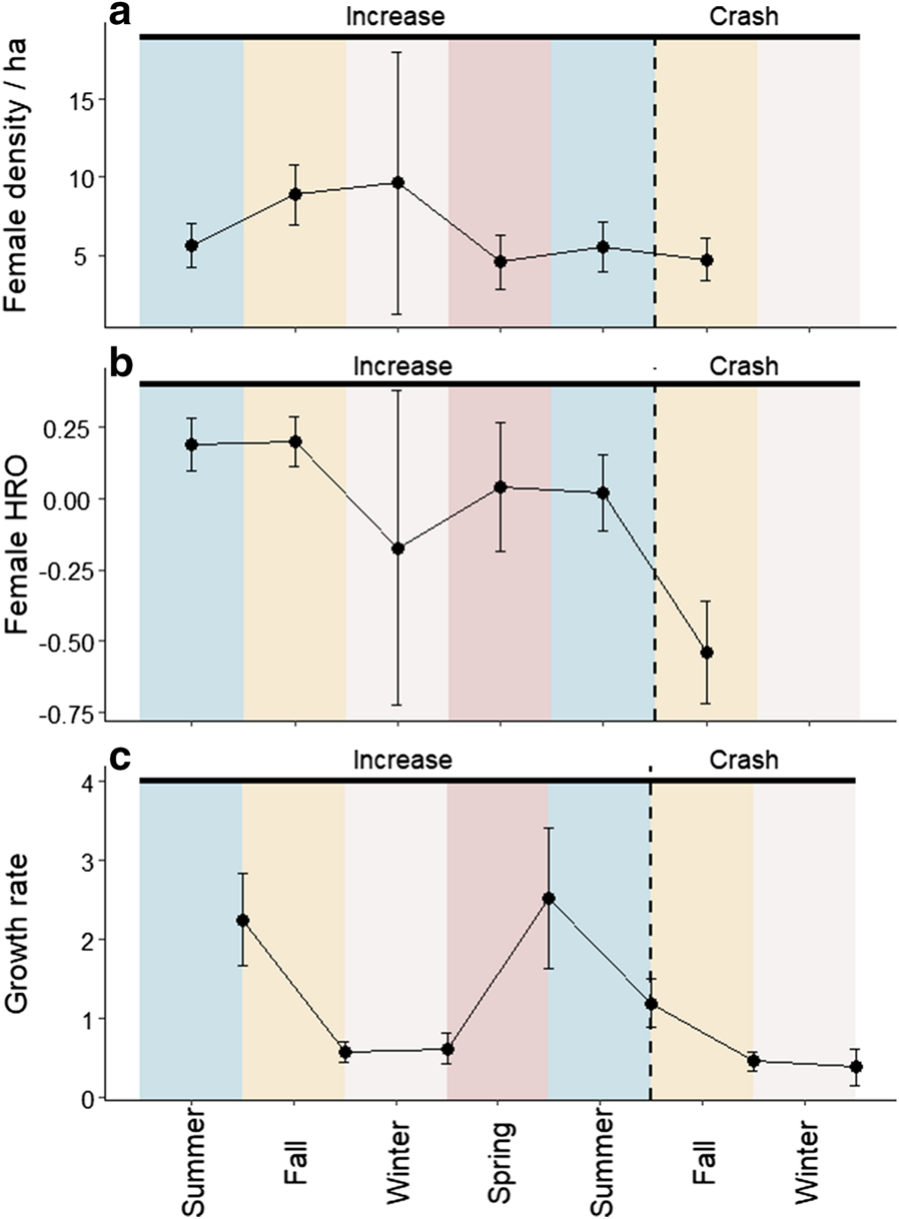
Female density per hectar (mean ± 2SE) (**a**), residual female home range overlap (mean ± 2SE) (**b**), and population growth rate (mean ± 2SE) (**c**) across seasons from summer 2013 (June–August) to Winter 2014–2015 (December–February). Due to too few captures, we were not able to estimate female density and consequently home range overlap after November 2014

There was both a seasonal- and phase-dependent effect on female home range overlap, as the best model (Female home range overlap = *f* (phase + season)) accounted for 99% of the AICc-weights (Table 2). Female density did not decrease dramatically in the beginning of the crash phase (Fig. 3a). Female home range overlap was highest in the first summer/fall of the increase phase, i.e. in 2013, somewhat lower during the second increase year and lowest during the crash phase, in the fall of 2014 (Fig. 3b). The low home range overlap in fall 2014 was not due to the estimates being based on only 2 months’ data as including December in the fall 2014 estimates did not change it (mean residual home range overlap ± SE for Oct–Nov fall 2014 = − 0.22 ± 0.07, for Oct–Dec fall 2014 = − 0.25 ± 0.08).

**Table 2:** Model selection results of linear mixed model explaining female bank vole home range overlap

Variables	AICc	ΔAICc	Weight	df	logLik
Phase + season	− 50.5	0	0.998	7	32.994
Phase	− 38	12.53	0.002	4	23.251
Season	− 31.5	19.02	0	6	22.293
Null	− 28.8	21.72	0	3	17.555

All model combinations are presented

There were effects of season and female home range overlap on the population growth rate (Table 3). Population growth rates were highest during the increase phase in spring and summer. However, growth rates decreased between summer and fall 2014 (Fig. 3c). Growth rates were low in fall and winter during both the increase and crash phases.

**Table 3:** Model selection results of linear mixed model explaining growth rate in bank voles

Variables	AICc	ΔAICc	Weight	df	logLik
HRO + season	154.9	0	0.543	7	− 69.727
Season	156.7	1.84	0.217	6	− 71.835
Phase + HRO + season	157.3	2.37	0.166	8	− 69.695
Phase + season	158.9	3.97	0.074	7	− 71.714
Phase + HRO	194.3	39.36	0	5	− 91.757
Phase	194.8	39.93	0	4	− 93.169
HRO	196.7	41.78	0	4	− 94.094
Null	200.5	45.55	0	3	− 97.081

All model combinations are presented. HRO = female home range overlap

**Fig. 4:**
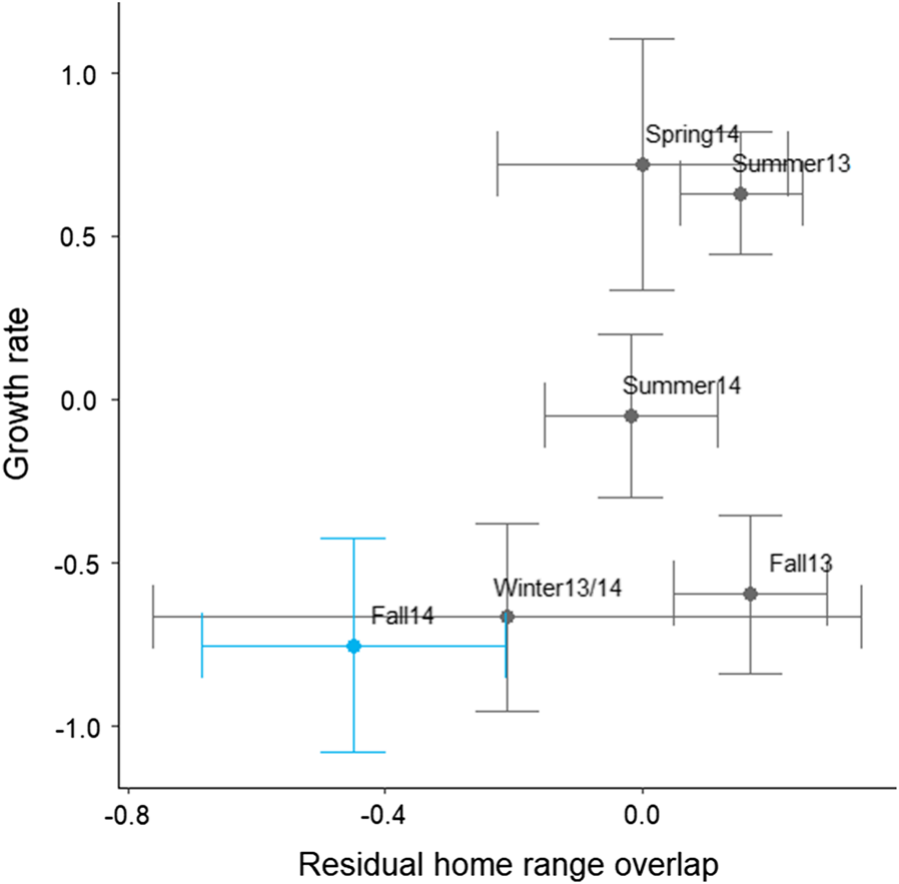
Correlation between population growth rate (mean ± 2SE) and residual female home range overlap (mean ± 2SE). Summer 13 = June–August 13, Fall 13 = September–November 13, Winter 13/14 = December–February 13/14, Spring 14 = March–May 14, Summer 14 = June–August 14, Fall 14 = October–November 14. Grey represent the increase phase of the cycle and blue represents the peak crash of the cycle

There was a correlation between female home range overlap and population growth rate (r_spear_ = 0.6). Generally, low female home range overlap corresponded with low population growth rates (Fig. 4). In the first fall, female home range overlap was high and associated with a population that had not yet crashed, even though the growth rate had dropped to a seasonal-dependent low.

## Discussion

Following a couple of decades without any high density peaks in Scandinavian vole populations [47], regular peaks, characteristic of cyclic vole populations, have occurred in the study area since 2007. In this study we were able to observe in detail a complete cycle including the increase and crash phase. The crash was observed as the extinction of trappable bank voles in our grids in May 2015. We analysed the data according to the framework for cyclic vole populations proposed by Andreassen et al. [5], in which phase-dependent social change was linked to population demography. We showed that population growth rate was associated with both season and female home range overlap, while female home range overlap was season- and phase-dependent. The increase phase was characterized by year round high female home range overlap and high population growth rates in spring/summer. The crash phase began when population density was still high but female home range overlap and population growth rate were low.

We chose to define two phases of the population cycle: an increase phase preceding and including the peak density observation in 2014, and a crash phase following the peak observation. Consequently there were two breeding seasons in the increase phase, with a non-breeding winter in-between during which the population density decreased. We did not consider this decrease as a crash, so much as a natural seasonal fluctuation in mortality combined with a lack of breeding. Most vole populations have been studied by trapping once or twice a year [47, 48], which does not allow such details in the population trajectories to be revealed. Obviously, high or peak vole densities, may occur 2 years in a row [49], which would extend the functional definition of the increase phase to 1–2 years, with natural seasonal fluctuations in densities. In contrast, we observed a crash phase characterized by a more consistent decrease in densities in fall/winter, which extended into the breeding season (summer 2015) and ended with the extinction of trappable animals. This is the typical crash phase of cyclic vole populations according to Hansson and Henttonen [50].

As predicted by Andreassen et al. [5] and Lambin and Krebs [7], adult females were most social during the increase phase, i.e. the female home range overlap were highest in this phase. Andreassen et al. [5] suggest that the individuals present at the start of the increase phase had survived the bottleneck of the previous cycle, i.e. the winter of the previous low phase, and that these individuals were presumed to be patchily located in high quality, core habitats [51]. Patchiness of resources is suggested to trigger the formation of groups in social females [13], leading to a higher breeding success and a higher population growth rate [35, 52]. In such a situation, the degree of kinship within the group could have a positive effect on recruitment and survival [7, 53, 54]. The more amicable behaviour of territorial females during the increase phase of the cycle may ultimately be due to kin structure [7, 55] and/or an adaptation to ample resources, thus making it more rewarding to use energy to survive and reproduce than to defend a territory against intruders [56].

Previous studies proposed that territorial behaviour should be more relaxed during the non-breeding season [57–59], and that winter aggregations and nest sharing were adaptations that may increase winter survival [60] by enhancing thermoregulation [37]. Although there was a large variation in female home range overlap during winter in our study, the mean female home range overlap decreased from fall to winter, indicating a higher degree of territoriality during winter. Food availability is an important factor affecting territoriality in females [13, 30, 31], and we know that it can be a limiting factor during winter [61, 62]. West and Dublin [60] proposed that food scarcity was the only reason to maintain territoriality in winter. Therefore, we suggest that in our study area territoriality is at least as strong in winter as during the breeding season due to the defence of possibly scarce and low-quality food resources.

Female home range overlap and population growth rate decreased dramatically from summer to fall in 2014, going from the increase to the crash phase, while the minimum number of voles known to be alive and the number of adult females remained quite high in fall 2014. This corresponded to the predictions of the framework described by Andreassen et al. [5] for a crash phase where female social system changes before estimates of abundance. In their framework the crash was initiated by predation, and that predation increased as a result of predators’ functional and numerical responses to high vole densities [63]. Further the framework suggests that as dominant males move over large areas and make risky movements to access females, they are more exposed to predation and consequently are the first individuals to be predated [64]. If the dominant males disappear, immigration of new males to vacant territories may reduce recruitment and the population growth rate due to infanticide [27, 32]. Although density can still be high at the beginning of this stage, the social system has been disrupted and contribute to further decrease in densities. Then the framework describes that females that have been exposed to infanticide and have lost their pups, tend to start moving around and become increasingly exposed to predation. In this situation, a more aggressive and territorial behaviour may be an adaptation to avoid infanticide, hostile individuals and predation. These intrinsic and extrinsic factors may lead to a population crash according to the framework described by Andreassen et al. [5]. In an individual-based model system, Radchuk et al. [26] confirmed that this combination of extrinsic (predation) and intrinsic (sociality and dispersal) factors could shape population cycles.

Alternatively, a crash could be initiated by limited food resources as a result of overexploitation during two high density summers. Bank voles have been found to lose weight and die when feeding on a green diet alone [65], so they may depend on specific high quality food resources for growth and survival (see also [66]). A shortage of seeds, for instance, could force adult females to become more territorial during the crash phase [11], as observed in our study. Home range overlap was lower during the crash phase than during the peak phase in the mast-induced cyclic yellow-necked mouse *Apodemus flavicollis* [8].

## Conclusions

Although the generality of the association we observed between changes in territorial behaviour and phase of the population cycles may be questioned because it is based on only one population cycle, our observations came from 24 populations across 3 transects covering a large area (113 km^2^ area between the most distant grids). Moreover, an association between social structure and population growth rates has been described for several species of rodents from the Cricetidae family, including the house mouse (*Mus* spp.) [67, 68], yellow-necked mouse [8], and *Myodes* and *Microtus* voles (see Andreassen et al. [5] and references therein). These species inhabit a variety of biomes and show population dynamics that vary from occasional outbreaks to more regular population peaks characteristics of population cycles. In all these situations, amicable behaviour has been associated with the increase phase of population density, often related to ample resources, while the decrease or crash phase of the fluctuations has been associated with disruption of the social system and a higher degree of territoriality. In contrast, the evidence that food, predation and/or kin structure shape changes in social behaviour, and in turn population dynamics, is equivocal.
